# Quenching of the red Mn^4+^ luminescence in Mn^4+^-doped fluoride LED phosphors

**DOI:** 10.1038/s41377-018-0013-1

**Published:** 2018-05-23

**Authors:** Tim Senden, Relinde J.A. van Dijk-Moes, Andries Meijerink

**Affiliations:** 1 Condensed Matter and Interfaces, Debye Institute for Nanomaterials Science, Utrecht University, P.O. Box 80000, 3508 TA Utrecht, The Netherlands; 2 Soft Condensed Matter, Debye Institute for Nanomaterials Science, Utrecht University, P.O. Box 80000, 3508 TA Utrecht, The Netherlands

## Abstract

Red-emitting Mn^4+^-doped fluorides are a promising class of materials to improve the color rendering and luminous efficacy of white light-emitting diodes (w-LEDs). For w-LEDs, the luminescence quenching temperature is very important, but surprisingly no systematic research has been conducted to understand the mechanism for thermal quenching in Mn^4+^-doped fluorides. Furthermore, concentration quenching of the Mn^4+^ luminescence can be an issue but detailed investigations are lacking. In this work, we study thermal quenching and concentration quenching in Mn^4+^-doped fluorides by measuring luminescence spectra and decay curves of K_2_TiF_6_:Mn^4+^ between 4 and 600 K and for Mn^4+^ concentrations from 0.01% to 15.7%. Temperature-dependent measurements on K_2_TiF_6_:Mn^4+^ and other Mn^4+^-doped phosphors show that quenching occurs through thermally activated crossover between the ^4^T_2_ excited state and ^4^A_2_ ground state. The quenching temperature can be optimized by designing host lattices in which Mn^4+^ has a high ^4^T_2_ state energy. Concentration-dependent studies reveal that concentration quenching effects are limited in K_2_TiF_6_:Mn^4+^ up to 5% Mn^4+^. This is important, as high Mn^4+^ concentrations are required for sufficient absorption of blue LED light in the parity-forbidden Mn^4+^ *d*–*d* transitions. At even higher Mn^4+^ concentrations (>10%), the quantum efficiency decreases, mostly due to direct energy transfer to quenching sites (defects and impurity ions). Optimization of the synthesis to reduce quenchers is crucial for developing more efficient highly absorbing Mn^4+^ phosphors. The present systematic study provides detailed insights into temperature and concentration quenching of Mn^4+^ emission and can be used to realize superior narrow-band red Mn^4+^ phosphors for w-LEDs.

## LEDs: Making white light more efficiently

The efficiency of white LEDs could be improved by considering the thermal properties of their coatings, say researchers in the Netherlands. White LED bulbs comprise a blue-emitting LED with a phosphorescent coating that converts some blue light to red and green, however conventional coatings waste energy by producing wavelengths beyond what the eye can see. Manganese-doped fluoride coatings, which are cheap and easy to make, show promise for reducing this energy loss. Tim Senden and co-workers at Utrecht University performed a detailed study of these coatings, exploring how their red-light emission depends on the manganese doping concentration and their temperature as the bulb warms up. Their findings provide the first clear explanation in terms of energy states for why emission tends to drop off with temperature, and will be useful for designing more efficient coatings.

## Introduction

White light-emitting diodes (w-LEDs) are the next-generation light sources for display and illumination systems because of their small size, high luminous efficacy, and long operation lifetime^1–5^. Conventional w-LEDs are composed of blue-emitting (In,Ga)N LEDs and green/yellow-emitting and orange/red-emitting phosphors that convert part of the blue LED emission^5–7^. Both phosphors are necessary to generate warm white light with a high color rendering index (CRI > 85). The typical red phosphors in w-LEDs are Eu^2+^-doped nitrides (e.g., CaAlSiN_3_:Eu^2+^)^4,8^. These phosphors exhibit high photoluminescence (PL) quantum efficiencies (QEs > 90%), but their use also has a serious drawback. The Eu^2+^ emission band is broad and extends into the deep red spectral region (*λ* > 650 nm) where the eye sensitivity is low. This causes the luminous efficacy of the w-LED to drop (reduced lumen/W output). A worldwide search is therefore aimed at finding efficient narrow-band red-emitting phosphors that can be excited by blue light. In this search, Mn^4+^-doped fluoride phosphors, such as K_2_SiF_6_:Mn^4+^ and K_2_TiF_6_:Mn^4+^, have recently attracted considerable attention^9–13^. Under blue light excitation, Mn^4+^-doped fluorides show narrow red line emission (*λ*_max_ ~ 630 nm) with high luminescence QEs^13–16^. Furthermore, they are prepared through low-cost, simple wet-chemical synthesis at room temperature^11,17^. These aspects make Mn^4+^-doped fluorides very promising red-emitting phosphors for developing energy-efficient high color-rendering w-LED systems^9^.

The application of Mn^4+^-doped fluoride phosphors in w-LEDs may, however, be hampered by thermal quenching of the Mn^4+^ luminescence. Thermal quenching of the phosphor luminescence is a serious issue, as it affects both the efficacy and color stability of the w-LED. In high-power w-LEDs, the temperature of the on-chip phosphor layer easily reaches 450 K. At these elevated temperatures, thermal quenching occurs for Mn^4+^-doped fluorides. The luminescence quenching temperature *T*_½_, the temperature at which the emission intensity is reduced to half of its maximum, is typically between 400 and 500 K^15,18,19^. Although the temperature dependence of the emission intensity has been measured for many Mn^4+^-doped fluorides, the understanding of the thermal quenching behavior is still limited. Most studies do not explain which process quenches the Mn^4+^ luminescence^13,20–23^. Moreover, the few reports that do propose a quenching mechanism disagree. Paulusz^15^ states that the luminescence of Mn^4+^-doped fluorides is quenched by thermally activated crossing of the Mn^4+ 4^T_2_ excited state and ^4^A_2_ ground state. In contrast, Dorenbos^24^ finds a relation between the quenching temperature and the energy of the F^−^ → Mn^4+^ charge-transfer (CT) state and therefore suggests that quenching involves crossover between the CT state and ^4^A_2_ ground state. This CT state crossover mechanism was also used by Blasse and our group to explain thermal quenching in Mn^4+^-doped oxides^25–27^. Finally, other reports claim that the quenching temperature increases if the radius of the cation substituted by Mn^4+^ becomes smaller^11,18^. A better understanding of the thermal quenching behavior is essential for developing Mn^4+^-doped fluoride phosphors with superior quenching temperatures, and thereby improving their potential for application in w-LEDs.

**Fig. 1:**
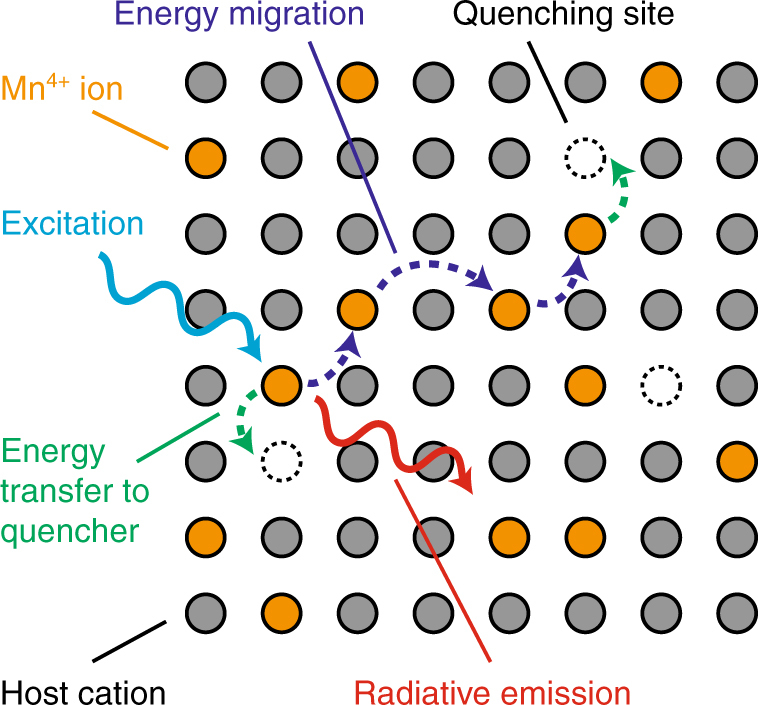
Concentration quenching for Mn^4+^ in crystals. At high Mn^4+^ doping concentrations the Mn^4+^ ions (orange) are in close proximity in the crystal lattice. If the Mn^4+^ ions are close together, energy transfer between Mn^4+^ ions (dark blue) causes the excitation to migrate through the crystal. Eventually, it may reach a quenching site such as a vacancy or impurity (dashed circle), where the excitation energy is lost as heat. This process competes with radiative emission (red) and reduces the luminescence efficiency

Besides thermal quenching, concentration quenching is an issue for the application of Mn^4+^-doped fluorides in w-LEDs. As the Mn^4+^ *d*–*d* transitions are parity-forbidden, high Mn^4+^ doping concentrations (e.g., 5 mol%) are required for sufficient absorption of the blue LED light^12^. At high dopant concentrations, energy migration among the Mn^4+^ ions can result in concentration quenching^26,28^, as is illustrated in Fig. 1. If the distance between the Mn^4+^ ions is small, excitation energy may efficiently migrate from one Mn^4+^ ion to another until it reaches a quenching site (defect or impurity ion), where the excitation energy is lost non-radiatively (as heat). Studies on concentration quenching in Mn^4+^-doped fluorides are limited. Several works have compared the luminescence properties of fluoride phosphors with varying Mn^4+^ concentrations, but do not measure the actual Mn^4+^ concentration in the phosphors by elemental analysis^29–33^. Determining the Mn^4+^ concentration is crucial, as often only a fraction of the Mn^4+^ ions is incorporated during the synthesis^19,34^. Reports that do perform elemental analysis study only a small range of Mn^4+^ doping concentrations and do not provide insight into the role of concentration quenching in Mn^4+^ doped fluorides^13,35,36^. An in-depth investigation of concentration quenching in Mn^4+^-doped fluorides is thus lacking, despite it being very important for the application of Mn^4+^-doped fluorides in w-LEDs.

In this work, we systematically investigate concentration quenching and thermal quenching in Mn^4+^-doped fluorides. The quenching is studied by measuring luminescence spectra and decay curves in the temperature range of 4 to 600 K for K_2_TiF_6_:Mn^4+^ phosphors with Mn^4+^ concentrations ranging from 0.01 to 15.7 mol% (actual Mn^4+^ concentration). The temperature-dependent luminescence measurements of K_2_TiF_6_:Mn^4+^ and other Mn^4+^-doped phosphors demonstrate that thermal quenching occurs because of thermally activated crossover from the ^4^T_2_ excited state to the ^4^A_2_ ground state. This insight into the quenching mechanism shows that the Mn^4+^ quenching temperature can be raised by finding fluoride hosts that have an increased Mn^4+ 4^T_2_ level energy. Concentration studies show that the luminescence QE of K_2_TiF_6_:Mn^4+^ is high, ~80%, for doping concentrations up to 5 mol% Mn^4+^. Concentration quenching is limited for these relatively high Mn^4+^ dopant concentrations. At even higher doping concentrations of >10 mol%, the QE of K_2_TiF_6_:Mn^4+^ falls below 60%. Luminescence decay curves indicate that the drop in QE can be attributed to an increased probability for direct energy transfer to quenching sites (e.g., defects, impurity ions, Mn^2+^, and Mn^3+^), the concentration of which increases with the Mn^4+^ concentration. The present results provide an improved understanding of thermal quenching and concentration quenching in Mn^4+^-doped solids and can be used to develop superior Mn^4+^-doped fluoride phosphors for w-LEDs.

## Materials and methods

### Synthesis and characterization of K_2_TiF_6_:Mn^4+^ phosphors

The K_2_TiF_6_:Mn^4+^ (*x*%) phosphors were synthesized according to the method of Zhu et al.^13^ For the synthesis of K_2_TiF_6_:Mn^4+^ (0.8%), 0.0488 g of K_2_MnF_6_ (prepared following refs. ^37,38^) was dissolved in 2.5 mL of a 40 wt% HF solution (Fluka, 40 wt% HF in water). Next, the obtained yellow-brown solution was mixed with 4.5730 g of K_2_TiF_6_ (Sigma-Aldrich, p.a.) and then stirred for 1 h at room temperature to form K_2_TiF_6_:Mn^4+^ crystals. The K_2_TiF_6_:Mn^4+^ phosphor was isolated by decanting the HF solution, washing twice with 15 mL of ethanol and then drying the phosphor for 7 h at 75 °C. The other K_2_TiF_6_:Mn^4+^ (*x*%) phosphors were prepared following the same procedure but using other amounts of K_2_MnF_6_ and K_2_TiF_6_ as to obtain different Mn^4+^ doping concentrations.

Powder X-ray diffraction (see Supplementary Figure S1) confirms that the K_2_TiF_6_:Mn^4+^ (*x*%) phosphors exhibit the hexagonal crystal structure of K_2_TiF_6_ up to the highest doping concentration of 15.7% Mn^4+^. Furthermore, no impurities of K_2_MnF_6_ or other crystal phases are observed in the diffraction patterns. Scanning electron microscopy (SEM) images show that most K_2_TiF_6_:Mn^4+^ phosphor particles are irregularly shaped and have sizes ranging from 1 to 200 µm (see Supplementary Figure S2a). Some particles have a hexagonal shape, in agreement with the hexagonal crystal structure of K_2_TiF_6_ (see Supplementary Figure S2b). Energy-dispersive X-ray (EDX) spectra (see Supplementary Figure S2c) confirm that the phosphor particles consist of potassium, titanium, fluorine, and manganese ions. The manganese dopant concentrations in the K_2_TiF_6_:Mn^4+^ phosphors were determined with inductively coupled plasma optical emission spectroscopy (ICP-OES). The ICP-OES measurements were performed on a Perkin-Elmer Optima 8300DV spectrometer (*λ*_em_ = 257.61 and 259.37 nm). For the ICP-OES analyses, the K_2_TiF_6_:Mn^4+^ phosphors were dissolved in aqua regia.

### Optical spectroscopy

PL measurements were performed on an Edinburgh Instruments FLS920 fluorescence spectrometer, except for the PL decay measurements between 300 and 600 K (see below). For recording excitation and emission spectra, we used a 450 W Xe lamp as excitation source and a Hamamatsu R928 photomultiplier tube (PMT) with a grating blazed at 500 nm for detection of emission. For PL decay measurements, excitation was done with a tunable optical parametric oscillator (OPO) Opotek Opolette HE 355II laser (pulse width 10 ns, repetition rate 10 Hz) and emission was detected with a Hamamatsu H74220–60 PMT. The PL decay curves between 300 and 600 K were recorded on a different setup, which had an Ekspla NT 342B OPO laser (pulse width 5 ns, repetition rate 10 Hz) as excitation source and a 0.55 m Triax 550 monochromator combined with a Hamamatsu H74220–60 PMT for detection of emission. All PL decay curves were obtained by multi-channel scaling (MCS) with a PicoQuant TimeHarp 260 computer card. The K_2_TiF_6_:Mn^4+^ phosphors were cooled down to 4 K with an Oxford Instruments liquid helium flow cryostat. For PL measurements between 300 and 600 K samples were heated in a Linkam THMS600 temperature controlled stage. The PL quantum efficiencies of the phosphors were determined with a calibrated home-built setup, which consisted of a 65 W Xe lamp, excitation monochromator, integrating sphere (Labsphere) and CCD camera (Avantes AvaSpec-2048).

## Results and discussion

### Luminescence of K_2_TiF_6_:Mn^4+^

**Fig. 2:**
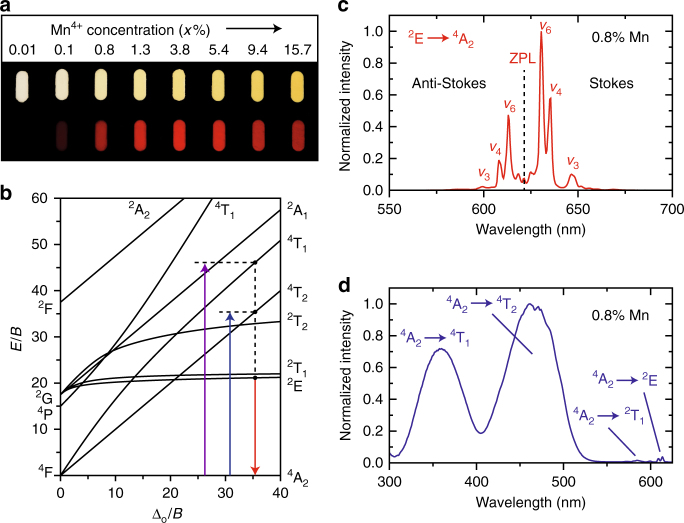
Mn^4+^ luminescence of K_2_TiF_6_:Mn^4+^. **a** Photographic image of K_2_TiF_6_:Mn^4+^ (*x*%) phosphors with *x* = 0.01, 0.1, 0.8, 1.3, 3.8, 5.4, 9.4, and 15.7. The phosphors have a white to yellow body color under ambient light (top) and show red Mn^4+^ luminescence under 365 nm UV illumination (bottom). **b** Tanabe−Sugano energy level diagram of the *d*^3^ electron configuration in an octahedral crystal field. The ^4^A_2_ → ^4^T_1_, ^4^A_2_ → ^4^T_2_, and ^2^E → ^4^A_2_ transitions of Mn^4+^ are indicated by the purple, blue and red arrows, respectively. Note that the excitation transitions are displaced for clarity. For a specific coordination all transitions take place around the same crystal field Δ_O_. **c** Emission spectrum of K_2_TiF_6_:Mn^4+^ (0.8%) upon excitation with blue light (*λ*_exc_ = 450 nm). **d** Excitation spectrum of the red Mn^4+^ luminescence (*λ*_em_ = 630 nm) from K_2_TiF_6_:Mn^4+^ (0.8%). Spectra are recorded at ambient temperature

For our quenching studies, we examine the luminescence of K_2_TiF_6_:Mn^4+^ phosphors with a wide range of Mn^4+^ doping concentrations. A photographic image of the K_2_TiF_6_:Mn^4+^ (*x*%) phosphors is displayed in Fig. 2a. The Mn^4+^ doping concentrations *x* (molar percentages with respect to Ti^4+^) were determined by inductively coupled plasma optical emission spectroscopy (ICP-OES). The body color of K_2_TiF_6_:Mn^4+^ becomes more yellow with increasing Mn^4+^ concentration as a result of enhanced absorption in the blue. All of the investigated K_2_TiF_6_:Mn^4+^ phosphors exhibit bright red Mn^4+^ luminescence under UV photoexcitation.

Figure 2b depicts the Tanabe–Sugano energy level diagram of Mn^4+^ (3*d*^3^ electron configuration) in an octahedral crystal field^39,40^. The diagram gives the *d*^3^ energy levels as a function of the crystal field splitting Δ_O_. Due to its high effective positive charge, Mn^4+^ experiences a strong crystal field and therefore the ^2^E state is the lowest energy excited state. Hence, the emission spectrum of K_2_TiF_6_:Mn^4+^ (0.8%) is dominated by narrow red emission lines due to spin- and parity-forbidden ^2^E → ^4^A_2_ transitions, as can be seen in Fig. 2c. The other K_2_TiF_6_:Mn^4+^ (*x*%) phosphors exhibit similar emission spectra. As the potential energy curves of the ^2^E and ^4^A_2_ states are at the same equilibrium position, the ^2^E → ^4^A_2_ emission is characterized by narrow zero-phonon and vibronic emission lines. The potential energy curves of the ^2^E and ^4^A_2_ states are at the same equilibrium position because the ^2^E and ^4^A_2_ states originate from the same $t_{{\mathrm{2g}}}^3$ electron configuration^41^.

The ^2^E → ^4^A_2_ emission spectrum consists of a weak zero-phonon line (ZPL) at ~622 nm and more intense anti-Stokes and Stokes vibronic emissions (labeled *ν*_3_, *ν*_4_, and *ν*_6_) on the high and low energy sides of the ZPL, respectively^13,15^. The ZPL is very weak because Mn^4+^ is located on a site with inversion symmetry in K_2_TiF_6_:Mn^4+^. Due to the inversion symmetry, there are no odd-parity crystal field components to admix opposite parity states into the ^4^A_2_ and ^2^E states and, as a result, the ^2^E → ^4^A_2_ transition is electric dipole forbidden. The ^2^E → ^4^A_2_ transition can become partly allowed, however, by coupling with asymmetric vibrations that induce odd-parity crystal field components. The most intense lines in Fig. 2c are assigned to ^2^E → ^4^A_2_ transitions coupling with the asymmetric *ν*_3_, *ν*_4_, and *ν*_6_ vibrational modes (phonons) of the ${\mathrm{MnF}}_6^{2 - }$ group. Thermal population of phonons at room temperature allows coupling with *ν*_3_, *ν*_4_, and *ν*_6_ phonon modes in the ^2^E excited state (giving rise to the anti-Stokes lines), while transitions to these phonon modes in the ^4^A_2_ ground state can occur at all temperatures (Stokes lines).

Figure 2d displays the excitation spectrum of the red Mn^4+^ luminescence from K_2_TiF_6_:Mn^4+^. The two broad excitation bands correspond to spin-allowed ^4^A_2_ → ^4^T_1_ and ^4^A_2_ → ^4^T_2_ transitions (violet and blue arrows in Fig. 2b). In addition, some weak peaks are visible around 600 nm. These peaks are assigned to ^4^A_2_ → ^2^E and ^4^A_2_ → ^2^T_1_ transitions. The ^4^A_2_ → ^2^T_1_,^2^E transitions are spin-forbidden and therefore low in intensity compared to the spin-allowed ^4^A_2_ → ^4^T_1_,^4^T_2_ transitions.

### Temperature dependence of the Mn^4+^ luminescence

To study the thermal quenching of the Mn^4+^ emission, we measure the PL intensity and Mn^4+^ emission lifetime of K_2_TiF_6_:Mn^4+^ (0.01%) as a function of temperature between 4 and 600 K. We use a very low Mn^4+^ doping concentration of 0.01%, as for higher Mn^4+^ concentrations reabsorption of emission and energy transfer between Mn^4+^ ions can occur. These processes will influence (the temperature dependence of) the Mn^4+^ luminescence spectra and decay curves^6^. As a result, with a high concentration of Mn^4+^ ions, the observations may not reflect the intrinsic thermal quenching properties of Mn^4+^.

**Fig. 3:**
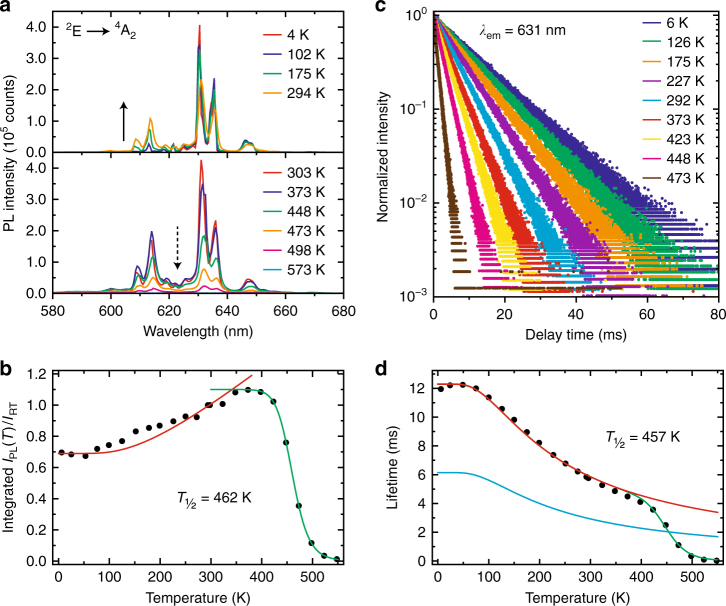
Temperature dependence of the Mn^4+^ luminescence from K_2_TiF_6_:Mn^4+^ (0.01%). **a** Emission spectra (*λ*_exc_ = 450 nm) of K_2_TiF_6_:Mn^4+^ (0.01%) at various temperatures between 0 and 600 K. **b** Integrated PL intensity of K_2_TiF_6_:Mn^4+^ (0.01%) as a function of temperature. The integrated PL intensity *I*_PL_ is scaled to the integrated PL intensity at room temperature *I*_RT_. The red and green lines represent fits to Eqs. 6 and 7, respectively. **c** PL decay curves of the Mn^4+^ emission from K_2_TiF_6_:Mn^4+^ (0.01%) at various temperatures between 0 and 600 K (*λ*_exc_ = 450 nm and *λ*_em_ = 631 nm). **d** Temperature dependence of the Mn^4+^ emission lifetime for K_2_TiF_6_:Mn^4+^ (0.01%). The red and green lines represent fits to Eqs. 4 and 8, respectively. The cyan line gives the fit for Eq. 4 (red line) divided by two

Figure 3a shows emission spectra of K_2_TiF_6_:Mn^4+^ (0.01%) at various temperatures between 4 and 600 K. At 4 K the Mn^4+ 2^E → ^4^A_2_ emission spectrum consists of zero-phonon and Stokes vibronic lines. Upon raising the temperature, phonon modes are thermally populated and anti-Stokes emission lines appear (solid arrow in Fig. 3a). With the appearance of anti-Stokes lines, the relative intensity of the Stokes emission decreases between 4 and 300 K. Above 400 K the intensities of both the anti-Stokes and Stokes emission lines begin to decrease (dashed arrow in Fig. 3a), which indicates the onset of non-radiative transitions from the ^2^E excited state. The luminescence is quenched at 600 K. From the measurements, we obtain the temperature dependence of the integrated PL intensity (*I*_PL_) relative to the integrated PL intensity at room temperature (*I*_RT_) (Fig. 3b). The PL intensity of K_2_TiF_6_:Mn^4+^ (0.01%) gradually increases between 4 and 350 K but then rapidly drops due to the onset of non-radiative transitions (luminescence quenching).

An alternative method to determine the luminescence quenching temperature is by measuring luminescence decay times. Figure 3c shows a selection of PL decay curves of K_2_TiF_6_:Mn^4+^ (0.01%) measured between 4 and 600 K. The decay of the Mn^4+^ emission is single exponential and becomes faster with increasing temperature. The PL decay time is on the order of milliseconds, which is expected as the transition between the ^2^E and ^4^A_2_ states is both parity- and spin-forbidden. In Fig. 3d, the Mn^4+^ emission lifetime (determined from single exponential fitting) is plotted as a function of temperature. The lifetime shows a steady decrease, starting above 50 K. The decrease levels off between 300 and 400 K but then shows a rapid decrease above 400 K.

The temperature dependences observed in Fig. 3b and d are quite exceptional. For most luminescent materials, the PL intensity and lifetime are relatively constant with temperature and both begin to decrease once thermal quenching sets in^6,42,43^. The PL intensity of K_2_TiF_6_:Mn^4+^, however, rises by 40% between 4 and 350 K while the lifetime decreases before thermal quenching takes place. To understand this peculiar temperature dependence, we first discuss how the radiative decay rate of the ^2^E state changes with temperature. The ^2^E → ^4^A_2_ emission of K_2_TiF_6_:Mn^4+^ mainly consists of anti-Stokes and Stokes vibronic emissions (Fig. 2c). Their transition probabilities increase with phonon population. The population of phonon modes is given by the phonon occupation number *n*, which increases with temperature according to^41^:

1 $$n = \frac{1}{{\exp \left( {hv/k_{\mathrm{B}}T} \right) - 1}}$$

where *k*_B_ is the Boltzmann constant and *hν* is the energy of the phonon coupling to the ^2^E → ^4^A_2_ transition. The transition probabilities *P*_R_ of the anti-Stokes and Stokes vibronics scale with *n* by:

2 $${\mathrm{Anti-Stokes}}:\; P_{\mathrm{R}}\left( T \right) = P_{\mathrm{R}}\left( 0 \right)\left[ n \right]$$

3 $${\mathrm{Stokes}}:\;P_{\mathrm{R}}\left( T \right) = P_{\mathrm{R}}\left( 0 \right)\left[ {n + 1} \right]$$

where *P*_R_(0) is the transition probability at *T* = 0 K. As the radiative lifetime *τ*_R_ is proportional to 1/[*P*_R_(anti-Stokes) + *P*_R_ (Stokes)], it follows from Eqs. 1–3 that:

4 $$\tau _{\mathrm{R}}\left( T \right) = \frac{{\tau _{\mathrm{R}}\left( 0 \right)}}{{{\mathrm{coth}}(hv/2k_{\mathrm{B}}T)}}$$

Here, *τ*_R_(0) is the radiative lifetime at *T* = 0 K. In Fig. 3d,Eq. 4 (red line) has been plotted for *τ*_R_(0) = 12.3 ms and *hν* = 216 cm^−1^ (phonon energy of the intense *ν*_6_ mode emission). Equation 4 accurately describes the measured temperature dependence of the Mn^4+^ emission lifetime up to 375 K, confirming that the decay of the ^2^E state is mainly radiative up to this temperature. The radiative lifetime of the Mn^4+^ emission shortens with temperature due to thermal population of odd-parity vibrational modes at higher temperatures.

Next, we investigate the increase in PL intensity between 4 and 350 K. The PL intensity *I*_PL_ equals the product of the PL QE and number of absorbed photons (as *I*_PL_ scales with the number of absorbed photons, the excitation wavelength can have a large influence on the temperature dependence observed for *I*_PL_; see Supplementary Information). The PL QE *η* of K_2_TiF_6_:Mn^4+^ can be expressed as:

5 $$\eta = \frac{{\gamma _{\mathrm{R}}}}{{\gamma _{\mathrm{R}} + \gamma _{{\mathrm{NR}}}}}$$

where *γ*_R_ and *γ*_NR_ are the radiative and non-radiative decay rates of the emitting ^2^E state, respectively. The results in Fig. 3d show that the decay of the ^2^E state is mainly radiative up to 375 K, so we can assume that *γ*_NR_ is negligible between 0 and 350 K. The value for *η* is therefore approximated as a constant close to unity between 0 and 350 K. On the other hand, the ^4^A_2_ → ^4^T_2_ absorption will change with temperature. Like the ^2^E → ^4^A_2_ transition, the ^4^A_2_ → ^4^T_2_ transition is electric dipole (parity) forbidden and gains intensity by coupling with vibrations (for more details on the vibronic structure of the ^4^A_2_ → ^4^T_2_ excitation band, see refs. ^15,16,44^). As a result, the PL intensity *I*_PL_ will scale with temperature as^20,41,45^:

6 $$I_{{\mathrm{PL}}}\left( T \right) = I\left( 0 \right){\mathrm{coth}}\left( {\frac{{hv}}{{2k_{\mathrm{B}}T}}} \right)$$

with *I*(0) being the PL intensity at *T* = 0 K. The results in Fig. 3b show that the increase in PL intensity between 4 and 350 K follows the temperature dependence given by Eq. 6. This confirms that the higher PL intensity at 350 K is due to a stronger absorption of excitation light. An increase in PL intensity between 4 and 350 K due to enhanced absorption is observed for all investigated Mn^4+^ doping concentrations (see Supplementary Information). Although the temperature dependence of the PL intensity follows Eq. 6, there is deviation between the fit of Eq. 6 and the measured data (see red line in Fig. 3b). The model of Eq. 6 is simple and does not take into account the shift and broadening of the ^4^A_2_ → ^4^T_2_ absorption band with temperature. Both these effects also influence the temperature dependence of the PL intensity, and this can explain the deviation between the model and the experimental data. Including the effect of a shift and broadening of the ^4^A_2_ → ^4^T_2_ band on the absorption strength is complex and will not aid a more accurate determination of *T*_½_.

Above 400 K the PL intensity of K_2_TiF_6_:Mn^4+^ (0.01%) begins to decrease due to the onset of non-radiative transitions (Fig. 3a, b). The non-radiative decay probability rapidly increases with temperature above 400 K and as a result the luminescence is quenched, with no emission intensity remaining at 600 K. The quenching temperature *T*_½_ is determined to be 462 K. The Mn^4+^ emission lifetime also rapidly decreases once thermal quenching sets in (Fig. 3d). Above 400 K the Mn^4+^ emission lifetime is shorter than the radiative lifetime *τ*_R_ predicted by Eq. 4 (red line). The lifetime shortens because of an additional thermally activated non-radiative contribution to the decay of the ^2^E state. From the temperature dependence of the lifetime, *T*_½_ can be determined by locating the temperature at which the lifetime has decreased to half of its radiative lifetime value. To estimate *T*_½_, we divide the value from the fit of Eq. 4 for *τ*_R_ by a factor of 2 (Fig. 3d, cyan line). The cyan line crosses the data points at 457 K. This value for *T*_½_ is very close to the *T*_½_ of 462 K obtained from the PL intensity measurements.

Thermal quenching can be described as a thermally activated process with an activation energy Δ*E*. The activation energy is obtained by fitting a modified Arrhenius equation to the temperature dependence of the PL intensity *I*_PL_ between 350 and 600 K^43,46^:

7 $$I_{{\mathrm{PL}}}\left( T \right) = \frac{{I\left( 0 \right)}}{{1 + A \times {\mathrm{exp}}\left( { - \Delta E/k_{\mathrm{B}}T} \right)}}$$

In Eq. 7, *I*(0) is the maximum PL intensity, *k*_B_ is the Boltzmann constant and *A* is a rate constant for the thermal quenching process. The best fit to Eq. 7 (green line in Fig. 3b) gives an activation energy ∆*E* of 9143 cm^−1^ and a rate constant *A* of 2.5 × 10^12^. We can also determine ∆*E* by fitting the temperature dependence of the Mn^4+^ emission lifetime *τ*(*T*) to the following expression^47^:

8 $$\tau \left( T \right) = \frac{{\tau _{\mathrm{R}}\left( T \right)}}{{{\mathrm{1}} + \left( {\frac{{\tau _{\mathrm{R}}\left( T \right)}}{{\tau _{{\mathrm{NR}}}}}} \right){\mathrm{exp}}\left( { - \Delta E/k_{\mathrm{B}}T} \right)}}$$

Here, 1/*τ*_NR_ is the non-radiative decay rate and *τ*_R_(*T*) is the radiative lifetime as described by Eq. 4 with *τ*_R_(0) = 12.3 ms and *hν* = 216 cm^−1^. We fit Eq. 8 to the Mn^4+^ emission lifetimes (green line in Fig. 3d) and find an activation energy ∆*E* of 7100 cm^−1^ and a prefactor 1/*τ*_NR_ of 1.5 × 10^12^ s^−1^. On the basis of the two similar values for ∆*E*, we conclude that the activation energy of the thermal quenching process is ~8000 cm^−1^. The rate constants *A* and 1/*τ*_NR_ should be approximately equal to the vibrational frequencies of the ${\mathrm{MnF}}_6^{2 - }$ group. The *ν*_6_ vibrational mode has a frequency of 6.5 × 10^12^ s^−1^, close to the rate constants found by fitting the data to Eqs. 7 and 8. The variation in activation energy values and prefactors can be explained by the fact that thermal quenching is not a simple thermally activated process. Struck and Fonger have shown that the temperature dependence of a non-radiative process is accurately described by considering ground and excited state vibrational wave function overlap^46,48^. According to the Struck–Fonger model, the non-radiative process occurs through tunneling (crossover) from a vibrational level of the excited state to a high vibrational level of the ground state. The tunneling rate, i.e., the non-radiative decay rate, depends on the wave function overlap of the vibrational levels involved. The tunneling rate will be faster for a larger overlap between the wave functions and when the vibrational levels are in resonance. For the present discussion, analysis of the data using complex models such as the Struck–Fonger model is not relevant, but it is important to realize that the Struck–Fonger model gives a more correct description of the actual quenching process.

### Thermal quenching in Mn^4+^-doped fluorides

To obtain insight into the thermal quenching of Mn^4+^ luminescence, we will discuss four possible quenching processes: (1) multi-phonon relaxation, (2) thermally activated photoionization, (3) thermally activated crossover via the F^−^ → Mn^4+^ charge-transfer (CT) state, and (4) thermally activated crossover via the Mn^4+ 4^T_2_ excited state.

**Fig. 4:**
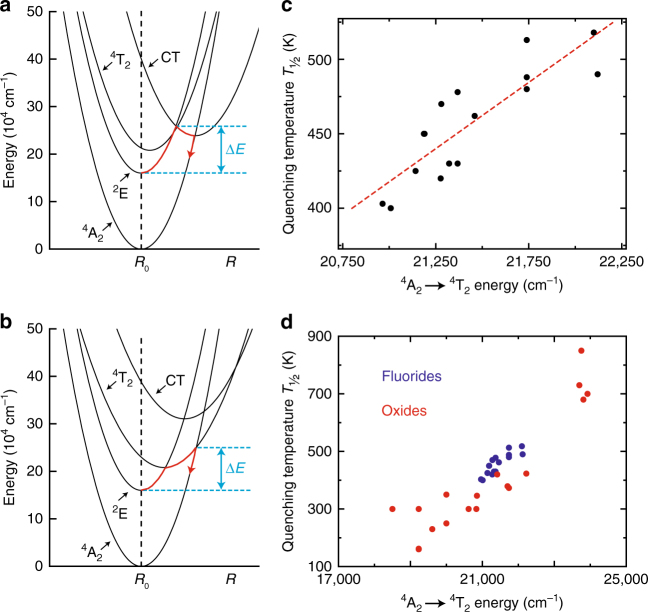
Thermal quenching in Mn^4+^-doped fluorides. **a**, **b** Configuration coordinate diagrams showing luminescence quenching due to **a** thermally activated crossover via the F^−^ → Mn^4+^ charge-transfer (CT) state and **b** thermally activated crossover via the Mn^4+ 4^T_2_ excited state. **c** Quenching temperature *T*_½_ of Mn^4+^-doped fluoride phosphors as a function of the ^4^A_2_ → ^4^T_2_ transition energy. The red dashed line is a linear fit to the data points. **d** Quenching temperature *T*_½_ of Mn^4+^-doped fluorides (blue dots) and Mn^4+^-doped oxides (red dots) as a function of the ^4^A_2_ → ^4^T_2_ transition energy

In the configurational coordinate diagram, the parabolas of the Mn^4+ 2^E and ^4^A_2_ states do not cross and luminescence quenching by crossover from the ^2^E to the ^4^A_2_ states is not possible (Fig. 4a). The ^4^A_2_ ground state may however be reached by multi-phonon relaxation. In Mn^4+^-doped fluorides more than 30 phonons of ~500 cm^−1^ are needed to bridge the energy gap between the ^2^E and ^4^A_2_ states^49^. For such high numbers of phonons (*p* > 30), it is unrealistic that non-radiative multi-phonon relaxation is responsible for thermal quenching (see Supplementary Information for a more detailed discussion). Alternatively, the thermal quenching can be due to thermally activated photoionization of an electron from the Mn^4+ 2^E state to the fluoride host conduction band. Thermally activated photoionization typically quenches the emission from a luminescent center if the emitting state is close in energy to the host conduction band^26,50^. In density functional theory (DFT) calculations, large band gaps of around 8 eV have been found for fluoride hosts like K_2_SiF_6_ and K_2_TiF_6_^51,52^. It is therefore expected that the Mn^4+ 2^E state is well below the host conduction band levels. Based on this, we conclude that thermal quenching in Mn^4+^-doped fluorides is not caused by thermally activated photoionization. However, more evidence is necessary to exclude this quenching mechanism. Photoconductivity measurements on Mn^4+^ phosphors at elevated temperatures need to be performed to provide convincing evidence for a possible role of photoionization in the thermal quenching of Mn^4+^ emission.

Thermal quenching in Mn^4+^-doped fluorides has been suggested to occur by thermally activated crossover via the Mn^4+ 4^T_2_ state or the F^−^ → Mn^4+^ charge-transfer (CT) state^15,24,26^. Both these states are displaced relative to the potential curve of the ^4^A_2_ ground state (Fig. 4a, b). Hence, the ^4^T_2_ and CT state parabolas cross the ^4^A_2_ ground state parabola. The difference between the potential curve equilibrium positions is given by the offset ∆*R* = *R*_0_′ − *R*_0_. By using the energies of the ^4^A_2_ → ^2^E, ^4^A_2_ → ^4^T_2_ and ^4^A_2_ → CT transitions in K_2_TiF_6_:Mn^4+^ (Fig. 2d and ref. ^13^) and assuming specific offsets ∆*R* for the ^4^T_2_ and CT states, we can construct the diagrams in Fig. 4a and b, where non-radiative relaxation occurs either via (a) the crossing of the CT and ^4^A_2_ states or (b) the crossing of the ^4^T_2_ and ^4^A_2_ states. The offset of the CT state is typically larger than the offset of the ^4^T_2_ state. Note that the diagrams in Fig. 4a and b are schematic configuration coordinate diagrams to illustrate the different quenching mechanisms.

In Fig. 4a, the CT state has a larger offset ∆*R* than the ^4^T_2_ state, which causes the CT parabola to cross the ^4^A_2_ parabola at lower energies than the ^4^T_2_ parabola. Thermal activation over the energy barrier ∆*E* will allow crossover from the ^2^E state into the CT state followed by non-radiative relaxation to the ground state via the crossing of the CT and ^4^A_2_ parabolas. Alternatively, thermal quenching of the Mn^4+^ luminescence may be due to the mechanism depicted in Fig. 4b. Here, the CT state has a smaller offset ∆*R* compared to that shown in Fig. 4a, and its potential curve is therefore at higher energies. In addition, the ^4^T_2_ state has a slightly larger offset. As a result, the crossing of the ^4^T_2_ and ^4^A_2_ parabolas is now at a lower energy and non-radiative relaxation will proceed via the crossing of the ^4^T_2_ and ^4^A_2_ parabolas.

The activation energies ∆*E* in the configuration coordinate diagrams are ~8000 cm^−1^, similar to the ∆*E* values obtained from the temperature-dependent measurements. This indicates that both mechanisms in Fig. 4a, b can explain the thermal quenching of Mn^4+^ luminescence. To determine which of these two mechanisms is responsible for the luminescence quenching, we compare the quenching temperature *T*_½_ of K_2_TiF_6_:Mn^4+^ to the *T*_½_ of other Mn^4+^-doped materials. A relation between the quenching temperature and the energy of either the CT or ^4^T_2_ state in a variety of hosts will give insight. If quenching occurs by crossover from the CT state to the ^4^A_2_ state, *T*_½_ will be higher for Mn^4+^-doped solids with higher CT transition energies. In K_2_TiF_6_:Mn^4+^ and other Mn^4+^-doped fluorides the F^−^ → Mn^4+^ CT transition is at ~40,000 cm^−1^^13,15^. Mn^4+^-doped oxides have lower O^2−^ → Mn^4+^ CT transition energies of 30,000–35,000 cm^−1^ and are therefore expected to have lower *T*_½_ values than fluorides if quenching occurs by the mechanism in Fig. 4a^26,27,53,54^. Some Mn^4+^-doped oxides, however, have much higher quenching temperatures than Mn^4+^-doped fluorides. For example, Mg_4_GeO_6_:Mn^4+^, Mg_28_Ge_7.5_O_38_F_10_:Mn^4+^, and Mg_6_As_2_O_11_:Mn^4+^ have a *T*_½_ of ~700 K^55–57^, while K_2_TiF_6_:Mn^4+^ and other Mn^4+^-doped fluorides have a *T*_½_ of 400–500 K (see also Tables 1 and 2). No correlation is found between the Mn^4+^ luminescence quenching temperature and the energy of the CT transition (see Supplementary Information for an overview and a plot of quenching temperatures and CT energies). From this we conclude that thermal quenching in Mn^4+^-doped fluorides is not caused by thermally activated crossover from the F^−^ → Mn^4+^ CT state to the ^4^A_2_ ground state.

**Table 1:** Quenching temperature *T*_½_ (K) and ^4^A_2_ → ^4^T_2_ energy (cm^−1^) for Mn^4+^-doped fluoride materials

Host lattice	^4^A_2_ → ^4^T_2_ energy (cm^−1^)	*T*_½_ (K)	References
K_2_TiF_6_	21,459	462	This work
K_2_SiF_6_	22,099	518	This work
K_2_SiF_6_	22,120	490	15
K_2_GeF_6_	21,280	470	15
K_2_TiF_6_	21,190	450	15
K_2_TiF_6_	21,368	478	13
Na_2_SiF_6_	21,739	488	21
Rb_2_SiF_6_	21,739	480	18
Rb_2_TiF_6_	21,186	450	18
Rb_2_GeF_6_	21,739	513	60
Cs_2_GeF_6_	21,277	420	22
Cs_2_SiF_6_	21,368	430	22
Cs_2_HfF_6_	20,964	403	44
BaSiF_6_	21,322	430	23
BaSnF_6_	21,008	400	45
BaTiF_6_	21,142	425	61

**Table 2:** Quenching temperature *T*_½_ (K) and ^4^A_2_ → ^4^T_2_ energy (cm^−1^) for Mn^4+^-doped oxide materials

Host lattice	^4^A_2_ → ^4^T_2_ energy (cm^−1^)	*T*_½_ (K)	References
Mg_4_GeO_6_	23,697	730	55
Mg_28_Ge_7.5_O_38_F_10_	23,923	700	26,55,56
K_2_Ge_4_O_9_	21,739	373	62
K_2_Ge_4_O_9_ (site 1)	19,231	160	63
K_2_Ge4O_9_ (site 2)	21,700	379	63
Rb_2_Ge_4_O_9_ (site 1)	19,231	162	63
Rb_2_Ge_4_O_9_ (site 2)	20,850	346	63
Y_2_Mg_3_Ge_3_O_12_	23,753	850	64
La_3_GaGe_5_O_16_	21,413	420	65
La_2_ZnTiO_6_	19,608	230	66
La_2_MgTiO_6_	20,000	250	66
CaZrO_3_	18,500	300	25,26
Mg_6_As_2_O_11_	23,810	680	57
Y_3_Al_5_O_12_	20,619	300	67
Y_3_Al_5_O_12_	20,833	300	68
Sr_4_Al_14_O_25_	22,222	423	69
SrLaAlO_4_	19,231	300	53
LiGa_5_O_8_	20,000	350	70

Alternatively, thermal quenching of the Mn^4+^ luminescence can be caused by thermally activated crossover via the Mn^4+ 4^T_2_ excited state (Fig. 4b). To investigate the validity of this mechanism, we compare the *T*_½_ and ^4^A_2_ → ^4^T_2_ transition energies for K_2_TiF_6_:Mn^4+^ and a variety of other Mn^4+^-doped fluorides. From the literature and measurements on Mn^4+^ luminescence we have collected quenching temperatures and luminescence spectra, preferably for systems with low doping concentrations. Figures 2d and 3b show that K_2_TiF_6_:Mn^4+^ has a ^4^A_2_ → ^4^T_2_ energy of 21,459 cm^−1^ (maximum of the excitation band) and a *T*_½_ of 462 K. For K_2_SiF_6_:Mn^4+^, we measured a ^4^A_2_ → ^4^T_2_ energy of 22,099 cm^−1^ and a *T*_½_ of 518 K (Supplementary Figure S6, K_2_SiF_6_:Mn^4+^ BR301-C commercial phosphor from Mitsubishi Chemical, Japan). In Fig. 4c we plot the quenching temperature *T*_½_ against the ^4^A_2_ → ^4^T_2_ energy for K_2_TiF_6_:Mn^4+^, K_2_SiF_6_:Mn^4+^ and many other Mn^4+^-doped fluoride phosphors reported in the literature (displayed data also listed in Table 1). The data show that the *T*_½_ increases with the energy of the ^4^T_2_ state. The clear trend shows that the thermal quenching in Mn^4+^-doped fluorides is due to thermally activated crossover from the ^4^T_2_ excited state to the ^4^A_2_ ground state. Further confirmation for this quenching mechanism is provided by Mn^4+^ spectra measured at elevated temperatures (see Supplementary Information). Supplementary Figure S7 shows emission spectra of K_2_SiF_6_:Mn^4+^ at *T* = 573 and 673 K. At 573 K a broad ^4^T_2_ → ^4^A_2_ emission band is observed, which is almost completely quenched at 673 K. The initial rise of the ^4^T_2_ → ^4^A_2_ emission at elevated temperatures confirms thermal population of the ^4^T_2_ level, which eventually leads to thermal quenching of all Mn^4+^ emission via this state.

To investigate whether thermally activated crossing via the ^4^T_2_ state is also responsible for temperature quenching in Mn^4+^-doped oxides, we extend the data set of Fig. 4c with quenching temperatures reported for Mn^4+^-doped oxides. Figure 4d shows the quenching temperature *T*_½_ as a function of the ^4^A_2_ → ^4^T_2_ energy for the Mn^4+^-doped fluorides and oxides listed in Tables 1 and 2. The results show that *T*_½_ increases with the energy of the ^4^A_2_ → ^4^T_2_ transition. This indicates that the Mn^4+^ emission in fluorides and oxides are both quenched due to thermally activated crossover from the ^4^T_2_ excited state, and not the CT state as previously suggested in some reports^24–27^. The present results and analysis provide strong evidence that in many Mn^4+^ phosphors the thermal quenching mechanism involves thermally activated crossover via the ^4^T_2_ excited state. A contribution from other mechanisms cannot be ruled out and further research, for example, photoconductivity measurements and high pressure studies, can give additional information on the role of alternative quenching mechanisms.

As quenching occurs by thermally activated crossover via the ^4^T_2_ excited state, the quenching temperature *T*_½_ of the Mn^4+^ luminescence is controlled by the energy of the Mn^4+ 4^T_2_ state (the dependence of *T*_½_ on the energy of the ^4^T_2_ state is shown in Fig. 4c,d). In addition, the *T*_½_ of the Mn^4+^ luminescence depends on the offset ∆*R* between the ^4^T_2_ and ^4^A_2_ states, as ∆*R* also determines where the ^4^T_2_ and ^4^A_2_ states cross in the configuration coordinate diagram (Fig. 4a,b). The horizontal displacement of the ^4^T_2_ parabola will influence the quenching temperature. A variation in ∆*R* can explain the spread observed in the data of Fig. 4c and d. To investigate the variation in the offset ∆*R* for Mn^4+^-doped fluorides, we compare the bandwidth of the ^4^A_2_ → ^4^T_2_ excitation band in K_2_TiF_6_:Mn^4+^, K_2_SiF_6_:Mn^4+^ and Cs_2_HfF_6_:Mn^4+^ (see Supplementary Figure S9). The width of the ^4^A_2_ → ^4^T_2_ excitation band is controlled by the displacement of the ^4^T_2_ state and therefore gives a good indication of ∆*R*. Comparison of the ^4^A_2_ → ^4^T_2_ bandwidths shows that there is a variation in ∆*R* for Mn^4+^-doped fluorides. The variation in ∆*R* is small, however, compared to the differences in the ^4^T_2_ energy, and no correlation is observed between the spectral width and quenching temperatures. This indicates that the ^4^T_2_ level energy has the largest influence on the quenching temperature of Mn^4+^-doped fluorides.

Finally, in view of applications, it is interesting to see how we can control the ^4^T_2_ level energy (and thereby *T*_½_) through the choice of the host lattice. The energy of the Mn^4+ 4^T_2_ state depends on the crystal field splitting Δ_O_ (Fig. 2b), where Δ_O_ is typically larger for shorter Mn–F distances^44,58^. For Mn^4+^-doped fluorides the luminescence quenching temperature can therefore be raised by selecting host lattices with short M^4+^–F^−^ distances (see Supplementary Figure S10a). This is consistent with findings that *T*_½_ increases if the radius of the M^4+^ host cation decreases, as expected based on crystal field theory^11,18^. If, however, *T*_½_ is plotted against the M^4+^-ligand distance for both Mn^4+^-doped fluorides and Mn^4+^-doped oxides (see Supplementary Figure S10b), no correlation between *T*_½_ and the M^4+^-ligand distance is found. This shows that the crystal field splitting and ^4^T_2_ energy give a better indication of the quenching temperature for Mn^4+^-doped phosphors.

### Concentration quenching

In addition to insight into thermal quenching, concentration quenching in Mn^4+^-doped fluorides is important for application in w-LEDs. The weak parity-forbidden ^4^A_2_ → ^4^T_2_ absorption requires that commercial phosphors have high Mn^4+^ concentrations. If there is effective concentration quenching, the PL decay time and QE will decrease when the Mn^4+^ doping concentration is raised^26,28^. We therefore investigate concentration quenching in K_2_TiF_6_:Mn^4+^ by measuring the PL decay times and QEs of K_2_TiF_6_:Mn^4+^ phosphors with Mn^4+^ concentrations ranging from 0.01 to 15.7% Mn^4+^.

**Fig. 5:**
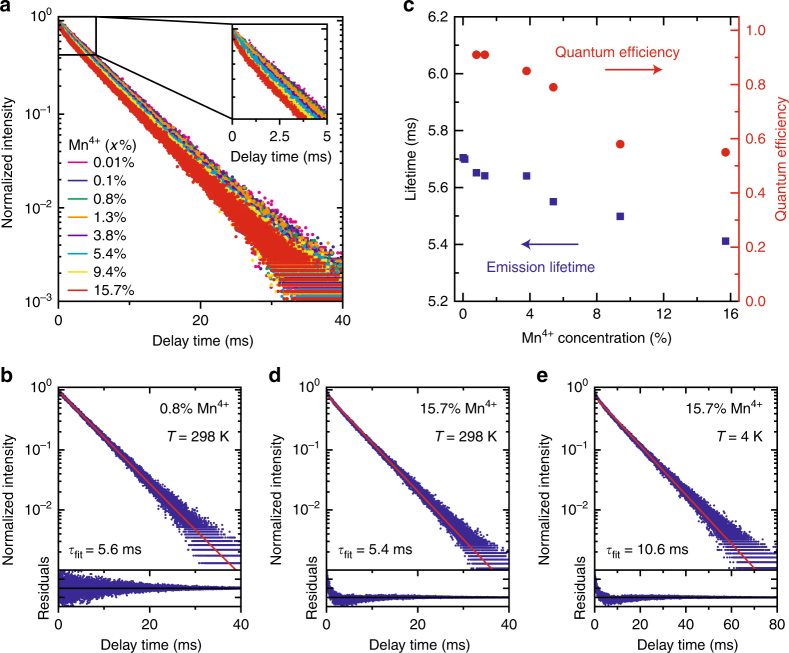
Luminescence decay and quantum efficiency of K_2_TiF_6_:Mn^4+^ as a function of the Mn^4+^ doping concentration. **a** Room-temperature PL decay curves of the Mn^4+^ emission from K_2_TiF_6_:Mn^4+^ (*x*%) for 0.01% (pink), 0.1% (blue), 0.8% (green), 1.3% (orange), 3.8% (purple), 5.4% (cyan), 9.4% (yellow), and 15.7% (red) Mn^4+^ (*λ*_exc_ = 450 nm and *λ*_em_ = 631 nm). **b** PL decay curve of K_2_TiF_6_:Mn^4+^ (0.8%) at *T* = 298 K. The decay time corresponding to the mono-exponential fit (red line) is 5.6 ms. The bottom panel shows the fit residuals. **c** Mn^4+^ emission lifetime (blue squares) and PL quantum efficiency (red dots) of K_2_TiF_6_:Mn^4+^ with different Mn^4+^ doping concentrations. **d**, **e** PL decay curves of K_2_TiF_6_:Mn^4+^ (15.7%) at **d** *T* = 298 K and **e** *T* = 4 K. The decay times corresponding to the mono-exponential fits (red lines) are 5.4 and 10.6 ms, respectively. The bottom panels show the fit residuals

Figure 5a presents room-temperature PL decay curves of the Mn^4+^ emission from K_2_TiF_6_:Mn^4+^ with increasing Mn^4+^ doping concentration *x*. It can be seen that the PL decay becomes slightly faster as the Mn^4+^ concentration increases. We analyze the decay dynamics by single exponential fitting of the PL decay curves. The fit for K_2_TiF_6_:Mn^4+^ (0.8%) is shown in Fig. 5b. The fit residuals (bottom panel) are random and the PL decay thus resembles a single exponential. This indicates that the decay of the ^2^E state is mainly radiative. Consequently, the K_2_TiF_6_:Mn^4+^ (0.8%) phosphor has a very high QE of 90%. Figure 5c gives an overview of the fitted decay times (blue squares) and QEs (red dots) of K_2_TiF_6_:Mn^4+^ with different Mn^4+^ concentrations. The emission lifetime barely shortens if the Mn^4+^ concentration is increased (5.7 ms for 0.01% Mn^4+^ to 5.4 ms for 15.7% Mn^4+^). This suggests that energy migration to quenching sites is inefficient in K_2_TiF_6_:Mn^4+^. To verify this, we look at the QE values obtained for the K_2_TiF_6_:Mn^4+^ (*x*%) phosphors. The QE remains above 80% for Mn^4+^ doping concentrations of 5% or less, which shows that concentration quenching is indeed limited up to a concentration of 5% Mn^4+^ ions. This result is important for applications in w-LEDs, as these high Mn^4+^ doping concentrations (e.g., 5 mol%) are required for sufficient absorption of the blue LED light in the parity-forbidden *d*–*d* transitions^12^.

For higher Mn^4+^ concentrations (*x* > 10%), non-radiative decay from the ^2^E excited state becomes stronger, however, and as a result the QE of K_2_TiF_6_:Mn^4+^ falls below 60% (Fig. 5c). The non-radiative decay is also visible in the PL decay curve of K_2_TiF_6_:Mn^4+^ (15.7%), shown in Fig. 5d. The decay is multi-exponential, which proves that with 15.7% Mn^4+^ the ^2^E state decays both radiatively and non-radiatively. The faster initial decay indicates that there is enhanced quenching by single-step energy transfer for Mn^4+^ ions close to a quencher. In case of energy migration, a faster decay is also expected for longer times after the excitation pulse. As this is not observed, the contribution of energy migration via many Mn^4+^ ions to quenching sites seems to be small.

To further investigate the role of energy migration in the concentration quenching of the Mn^4+^ emission, we measure a PL decay curve of K_2_TiF_6_:Mn^4+^ (15.7%) at *T* = 4 K, which is displayed in Fig. 5e. At *T* = 4 K energy migration among the Mn^4+^ ions (blue arrows in Fig. 1) will be hampered, as there is almost no spectral overlap between the Mn^4+ 2^E → ^4^A_2_ emission and ^4^A_2_ → ^2^E excitation lines (see Supplementary Figure S11). Hence, at 4 K non-radiative decay due to energy migration to quenching sites will be suppressed. The Mn^4+^ decay dynamics in Fig. 5e, however, show that the non-radiative decay is not suppressed at 4 K. The deviation from single exponential behavior is similar to that at 300 K. There is an initial faster decay (single-step energy transfer to quenching sites) followed by an exponential decay with a decay time very close to that measured for Mn^4+^ at low doping concentrations. This suggests that the decrease in QE at higher Mn^4+^ concentrations is not due to energy migration. The absence of strong concentration quenching by energy migration is confirmed by the thermal quenching behavior measured for the different Mn^4+^ concentrations. In Supplementary Figure S4, it can be seen that the luminescence quenching temperature is approximately the same for doping concentrations of 0.01% and 15.7% Mn^4+^, which shows that effects due to thermally activated energy migration (i.e., concentration quenching) are weak. Hence, we conclude that the non-radiative decay at high Mn^4+^ concentrations is not caused by energy migration. Inefficient energy migration can be understood based on the strongly forbidden character of the ^2^E → ^4^A_2_ transition. This allows only Mn^4+^–Mn^4+^ energy transfer via short range exchange interaction (see Supplementary Information for details).

We instead assign the non-radiative decay to direct transfer of excitation energy from Mn^4+^ ions to quenchers (green arrow in Fig. 1). This process can occur at all temperatures and becomes more efficient at higher Mn^4+^ dopant concentrations. With an increasing Mn^4+^ dopant concentration, the stress on the K_2_TiF_6_ lattice grows and as a result more crystal defects (i.e., quenchers) may be formed. In addition, Mn in different valence states (Mn^2+^ and Mn^3+^) may be incorporated at higher Mn^4+^ concentrations. Even if a very small fraction of Mn^4+^ ions has a different valence state than 4+, effective quenching can occur via metal-to-metal charge-transfer states or direct energy transfer. Consequently, the probability for energy transfer to quenchers increases, resulting in faster initial PL decay and lower QEs for K_2_TiF_6_:Mn^4+^ at high Mn^4+^ dopant concentrations. Optimized synthesis procedures to reduce quenchers (defects and impurity ions) are thus crucial for obtaining highly luminescent Mn^4+^-doped fluoride phosphors (see also recent work of Garcia-Santamaria et al.^59^ on concentration quenching in K_2_SiF_6_:Mn^4+^).

## Conclusions

Narrow-band red-emitting Mn^4+^ phosphors form an important new class of materials for LED lighting and displays. For these applications, it is important to understand and control the luminescence efficiency. We have therefore investigated quenching of the Mn^4+^ luminescence in Mn^4+^-doped fluorides by measuring the PL intensity and luminescence lifetimes of K_2_TiF_6_:Mn^4+^ between 4 and 600 K and for Mn^4+^ concentrations from 0.01 to 15.7%. Temperature-dependent measurements of the Mn^4+^ emission intensity and lifetime for K_2_TiF_6_:Mn^4+^ and other Mn^4+^-doped phosphors show that thermal quenching is caused by thermally activated crossover via the Mn^4+ 4^T_2_ excited state. As a result, the quenching temperature is higher in Mn^4+^-doped materials with higher ^4^T_2_ state energies. These findings can be used to engineer Mn^4+^-doped fluoride phosphors with higher quenching temperatures for application in high-power w-LEDs.

Furthermore, quantum efficiency and luminescence decay measurements for a wide range of Mn^4+^ doping concentrations show that no concentration quenching occurs up to 5% Mn^4+^ in K_2_TiF_6_:Mn^4+^. This is important for the application of Mn^4+^-doped materials in w-LEDs, as high Mn^4+^ doping concentrations (e.g., 5 mol%) are required for sufficient absorption of the blue LED light in the parity-forbidden Mn^4+^ *d*–*d* transitions. At very high Mn^4+^ doping concentrations (>10 mol%) the quantum efficiency of K_2_TiF_6_:Mn^4+^ decreases due to enhanced direct energy transfer from Mn^4+^ to quenching sites. Concentration quenching by Mn^4+^–Mn^4+^ energy migration is limited. To optimize the efficiency in highly doped Mn^4+^ phosphors, a synthesis procedure aimed at reducing quenching sites (defects, impurity ions, Mn^2+^, and Mn^3+^) will be crucial.

## Electronic supplementary material

Supplementary Information
