# Supplementary material for: Quenching of the red Mn4+ luminescence in Mn4+-doped fluoride LED phosphors
Source: Light Sci Appl. 2018 May 23;7:8. doi: 10.1038/s41377-018-0013-1 (PMC6106983; doi:10.1038/s41377-018-0013-1)
Supplement: Supplementary file 1 — Supplementary Information [file 41377_2018_13_MOESM1_ESM.pdf]

Supplementary Information

Quenching of the red Mn^{4+} luminescence in Mn^{4+}-doped fluoride LED phosphors

Tim Senden[a,*], Relinde J. A. van Dijk-Moes[b] and Andries Meijerink[a]

[a]Condensed Matter and Interfaces, Debye Institute for Nanomaterials Science, Utrecht University, P.O. Box 80000, 3508 TA Utrecht, The Netherlands. *Corresponding author; E-mail: t.senden@uu.nl; Telephone: +31 30 253 2214;
[b]Soft Condensed Matter, Debye Institute for Nanomaterials Science, Utrecht University, P.O. Box 80000, 3508 TA Utrecht, The Netherlands.

1. Powder X-ray diffraction

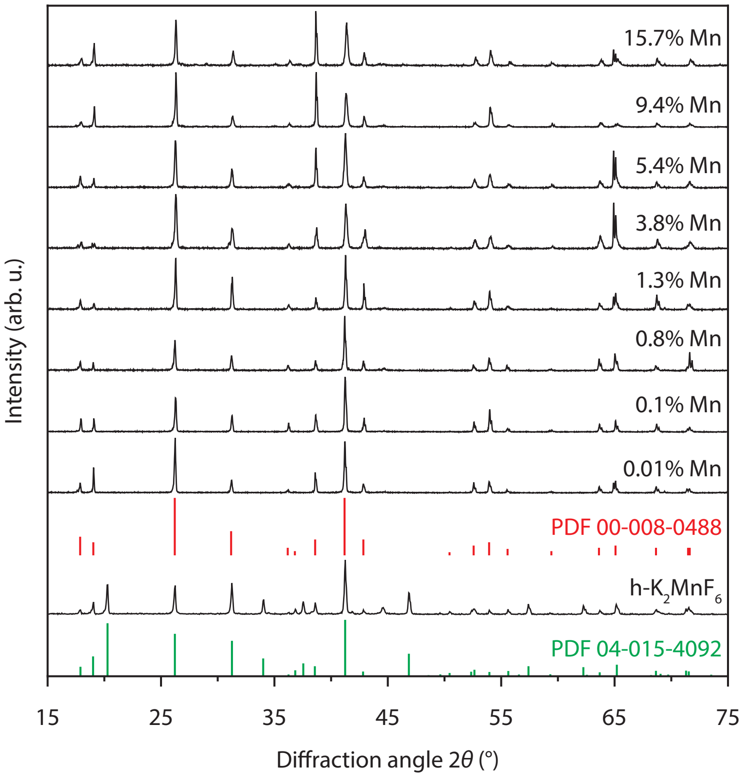

Figure S1 Powder X-ray diffraction (XRD) patterns of K_2MnF_6 and the K_2TiF_6:Mn^{4+} (x%) phosphors. The diffraction patterns are in agreement with the literature references for hexagonal phase K_2MnF_6 (PDF 04-015-4092) and K_2TiF_6 (PDF 00-008-0488).

2. Scanning electron microscopy and Energy-dispersive X-ray spectroscopy

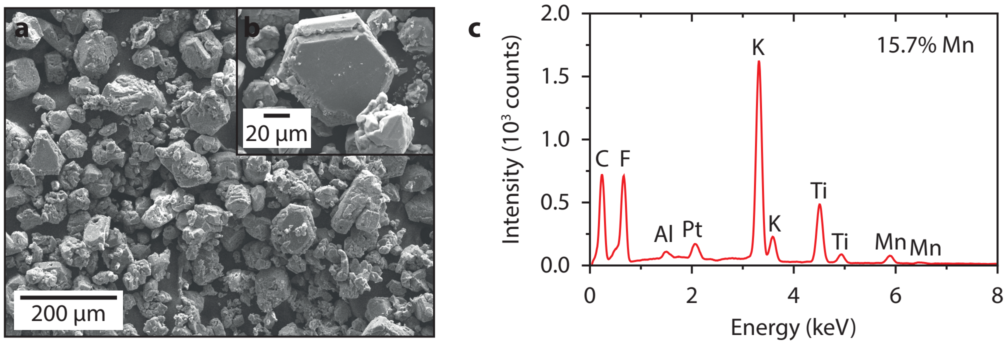

Figure S2 (**a**) Representative scanning electron microscopy (SEM) image of K_2TiF_6:Mn^{4+} (5.4%) phosphor. (**b**) SEM image of a single K_2TiF_6:Mn^{4+} (5.4%) phosphor particle displaying the hexagonal crystal structure of K_2TiF_6. (**c**) Energy dispersive X-ray (EDX) spectrum of K_2TiF_6:Mn^{4+} (15.7%). The peaks in the spectrum are labeled with their corresponding elements. The aluminium (Al) and carbon (C) peaks originate from the aluminium holder and carbon tape beneath the sample. The platinum (Pt) peak is due to the platinum layer sputtered onto the phosphor particles for the SEM-EDX measurements.

3. Influence of excitation wavelength on the observed thermal quenching behavior

The excitation wavelength used in photoluminescence (PL) measurements can have a large influence on the temperature dependence observed for the PL intensity I_{PL}. Because the Mn^{4+} excitation bands broaden and redshift with temperature (Figure S3a), exciting at the $^4A_2 \rightarrow {}^4T_2$ band maximum (450 nm) or $^4A_2 \rightarrow {}^4T_2$ band onset (405 nm) results in very different temperature dependences (Figure S3b). Consequently, different quenching temperatures $T_{½}$ and activation energies ΔE are obtained. As the true $T_{½}$ and ΔE are obtained when effects due to band broadening and shifting are minimized, the preferred excitation wavelength is at or close to the band maximum when measuring the temperature dependence for I_{PL}. However, even then a change in the emission intensity by variations in the absorption strength at the excitation wavelength can introduce an error in $T_{½}$. In general, temperature-dependent lifetime measurements provide a more reliable value for $T_{½}$[1].

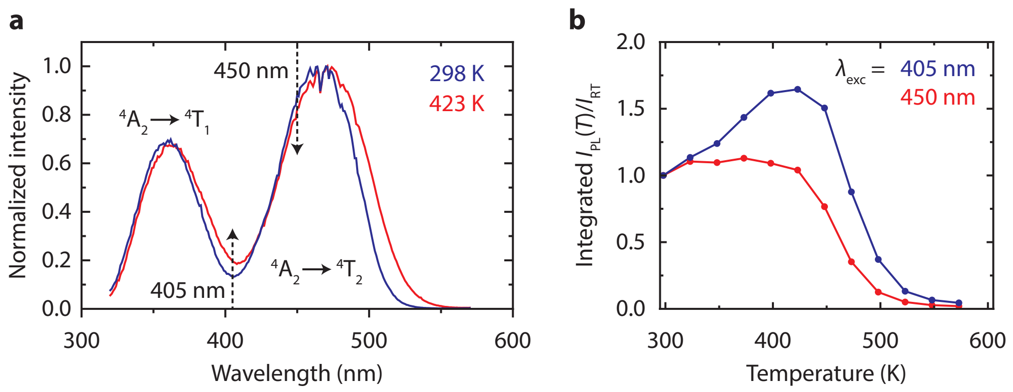

Figure S3 (**a**) Excitation spectra (λ_{em} = 631 nm) of $K_2TiF_6{:}Mn^{4+}$ (0.8%) at T = 298 K (blue) and 423 K (red). (**b**) Temperature dependence of the integrated PL intensity from $K_2TiF_6{:}Mn^{4+}$ (0.8%) for λ_{exc} = 405 nm (blue) and 450 nm (red). The PL intensity I_{PL} is given relative to the PL intensity at room temperature I_{RT}.

4. Temperature dependence of the PL intensity and lifetime for $K_2TiF_6{:}Mn^{4+}$ with different Mn^{4+} doping concentrations

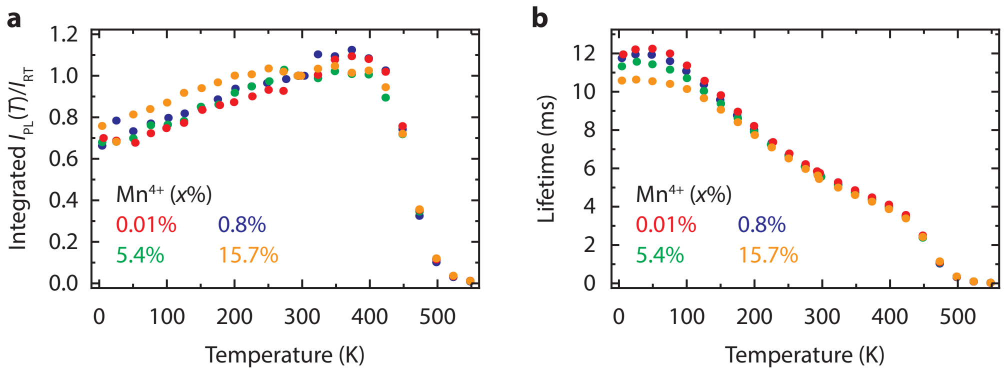

Figure S4 (**a**) Integrated PL intensity of $K_2TiF_6{:}Mn^{4+}$ (x%) as a function of temperature for x = 0.01 (red), 0.8 (blue), 5.4 (green) and 15.7% (orange). The integrated PL intensity I_{PL} is scaled to the integrated PL intensity at room temperature I_{RT}. (**b**) Temperature dependence of the Mn^{4+} emission lifetime for $K_2TiF_6{:}Mn^{4+}$ with 0.01% (red), 0.8% (blue), 5.4% (green) and 15.7% (orange) Mn^{4+}.

An increase in PL intensity between 4 and 350 K due to enhanced absorption is observed for all investigated Mn^{4+} doping concentrations. At high Mn^{4+} doping concentrations (i.e., 5.4 and 15.7%) the intensity increase is however smaller because of saturation effects (strong absorption of light at high dopant concentrations causes a sub-linear increase of the fraction of absorbed light with the transition probability)[1].

5. Quenching by multi-phonon relaxation

In the configurational coordinate diagram, the parabolas of the Mn^{4+} ^2E and $^4A_2$ states are at the same equilibrium position and luminescence quenching due to direct crossover from the ^2E excited state to the $^4A_2$ ground state is not possible (see configuration coordinate diagram in Figure 4a). The $^4A_2$ ground state may however be reached by multi-phonon relaxation. In some works on Mn^{4+}-doped oxides, thermal quenching of the Mn^{4+} luminescence was attributed to multi-phonon relaxation[2–5]. The temperature dependence of the luminescence intensity and emission lifetime was explained with a multi-phonon relaxation process involving more than 40 phonons. Also in Mn^{4+}-doped fluorides, a high number of phonons is necessary to reach the $^4A_2$ ground state via multi-phonon relaxation. In a fluoride, the maximum phonon energy ν_{max} is ~500 cm^{-1}, so around 32 high energy vibrations are needed to bridge the ~16000 cm^{-1} energy gap between the ^2E and $^4A_2$ states[6]. For such high numbers of phonons ($p > 30$), it is unrealistic that non-radiative multi-phonon relaxation is responsible for thermal quenching, as typically multi-phonon relaxation can only compete with radiative decay if the energy difference between the ground and excited state is equal to or less than 5 times the ν_{max} of the surrounding lattice. Moreover, if quenching would occur through multi-phonon relaxation, it is expected that the $T_{½}$ is relatively similar for the different Mn^{4+}-doped fluoride phosphors since all hexafluorometallates will have around the same maximum phonon energy ν_{max} (Ref. 7). There is however a large spread in the $T_{½}$ of Mn^{4+}-doped fluorides, varying from e.g., $T_{½}$ = 403 K in Cs_2HfF_6:Mn^{4+} to $T_{½}$ = 518 K in K_2SiF_6:Mn^{4+} (see also Table 1)[8]. Finally, the non-radiative decay rate due to multi-phonon relaxation increases with temperature as the non-radiative decay rate at low temperatures multiplied by $(n + 1)^p$ (Ref. 9). This implies that also at low temperatures multi-phonon relaxation should be effective as phonon emission can always occur. The observation of quantum efficiencies close to 100% at ambient temperature is not consistent with multi-phonon relaxation. We conclude that thermal quenching of the Mn^{4+} luminescence cannot be due to multi-phonon relaxation from the ^2E state.

6. Relation between quenching temperature and energy of Mn^{4+} charge-transfer transition

Figure S5 shows the luminescence quenching temperature of Mn^{4+}-doped fluorides and Mn^{4+}-doped oxides as function of the ligand-to-Mn^{4+} charge-transfer (CT) transition energy. The quenching temperatures and CT energies were collected from luminescence measurements presented in this work and the literature. The results in Figure S5 show that the Mn^{4+} luminescence quenching temperature and ligand-to-Mn^{4+} CT transition energy are not correlated. The data displayed in Figure S5 is also listed in Table S1. We want to note that for most Mn^{4+}-doped oxides the CT excitation band overlaps with the $^4A_2 \rightarrow {}^4T_1$ excitation band, which introduces an uncertainty in the CT energies reported in Table S1.

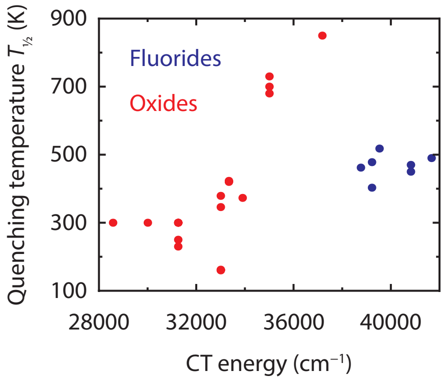

Figure S5 Quenching temperature $T_{½}$ of Mn^{4+}-doped fluorides (blue dots) and Mn^{4+}-doped oxides (red dots) as a function of the ligand-to-Mn^{4+} charge-transfer (CT) transition energy.

Table S1 Quenching temperature $T_{½}$ and charge-transfer (CT) transition energy of Mn^{4+}-doped phosphors.

Host lattice	CT energy (cm^{-1})	$T_{½}$ (K)	References
K_2TiF_6	38760	462	This work
K_2TiF_6	40816	450	10
K_2TiF_6	39216	478	11
K_2SiF_6	39526	518	This work
K_2SiF_6	41667	490	10
K_2GeF_6	40816	470	10
Cs_2HfF_6	39216	403	8
Mg_4GeO_6	35000	730	12
$Mg_{28}Ge_{7.5}O_{38}F_{10}$	35000	700	12–14
$K_2Ge_4O_9$	33898	373	15
$K_2Ge_4O_9$ (site 1)	33000	160	16
$K_2Ge_4O_9$ (site 2)	33000	379	16
$Rb_2Ge_4O_9$ (site 1)	33000	162	16
$Rb_2Ge_4O_9$ (site 2)	33000	346	16
$Y_2Mg_3Ge_3O_{12}$	37175	850	17
$La_3GaGe_5O_{16}$	33333	420	18
La_2ZnTiO_6	31250	230	19
La_2MgTiO_6	31250	250	19
$CaZrO_3$	30000	300	14,20
$Mg_6As_2O_{11}$	35000	680	21,22
$Y_3Al_5O_{12}$	31250	300	23
$Y_3Al_5O_{12}$	31250	300	24
$Sr_4Al_{14}O_{25}$	33333	423	25
$SrLaAlO_4$	28571	300	26

7. Luminescence spectra and thermal quenching for $K_2SiF_6:Mn^{4+}$

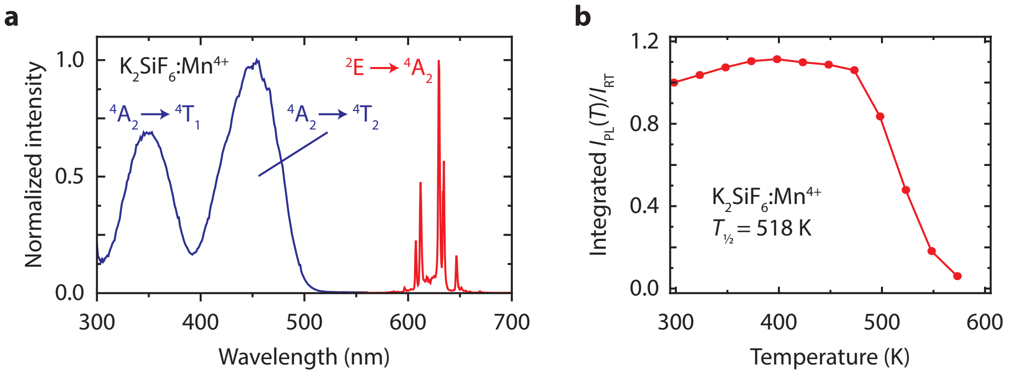

Figure S6 (**a**) Room-temperature PL excitation (blue, λ_{em} = 630 nm) and emission (red, λ_{exc} = 460 nm) spectra of $K_2SiF_6:Mn^{4+}$. The emission and excitation bands/lines are assigned to corresponding transitions in the d^3 Tanabe–Sugano diagram. (**b**) Integrated PL intensity of $K_2SiF_6:Mn^{4+}$ (λ_{exc} = 450 nm) as a function of temperature between 300 and 600 K. The PL intensity I_{PL} is given relative to the PL intensity at room temperature I_{RT}. The luminescence quenching temperature $T_{½}$ is 518 K.

8. Mn^{4+} luminescence spectra measured at elevated temperatures

Additional proof for thermal quenching by crossover via the $^4T_2$ state is obtained from Mn^{4+} luminescence spectra measured at elevated temperatures. Figure S7 shows emission spectra of $K_2SiF_6:Mn^{4+}$ (commercial phosphor) measured at T = 573 and 673 K. Besides the characteristic $^2E \rightarrow {}^4A_2$ emission lines, the luminescence spectra in Figure S7 exhibit some additional weak emission bands/lines at wavelengths shorter than 600 nm. These emissions are assigned to the Mn^{4+} $^4T_2 \rightarrow {}^4A_2$ and $^2T_1 \rightarrow {}^4A_2$ transitions. An excitation spectrum recorded for λ_{em} = 530 nm at T = 473 K (see Figure S8) confirms that the emission band centered at 530 nm is related to Mn^{4+}. The observation of Mn^{4+} $^4T_2 \rightarrow {}^4A_2$ emission in $K_2SiF_6:Mn^{4+}$ at 573 K shows that the $^4T_2$ excited state is indeed thermally populated at elevated temperatures, and consequently can play a role in the thermal quenching process. Upon further heating to 673 K, the intensities of the $^4T_2 \rightarrow {}^4A_2$ and $^2E \rightarrow {}^4A_2$ emissions decrease (green spectrum in Figure S7), and after cooling to 573 K, most of the $^4T_2 \rightarrow {}^4A_2$ and $^2E \rightarrow {}^4A_2$ emission intensity is regained (blue spectrum in Figure S7). These measurements indicate that the emission intensity decrease between 573 and 673 K is due to thermal quenching of both the $^4T_2 \rightarrow {}^4A_2$ and $^2E \rightarrow {}^4A_2$ emission, and not due to chemical degradation of the phosphor (the small difference in intensity at 573 K before and after heating to 673 K is however attributed to phosphor degradation). The fact that both the $^4T_2 \rightarrow {}^4A_2$ and $^2E \rightarrow {}^4A_2$ emission are quenched upon raising the temperature from 573 to 673 K shows that the loss in $^2E \rightarrow {}^4A_2$ emission intensity is not accompanied an increase in the $^4T_2 \rightarrow {}^4A_2$ emission intensity, as is sometimes observed for Cr^{3+} (isoelectronic with Mn^{4+})[27–29]. Instead, in $K_2SiF_6:Mn^{4+}$ the $^4T_2 \rightarrow {}^4A_2$ emission is quenched by non-radiative relaxation via the crossing of the $^4T_2$ state and $^4A_2$ ground state.

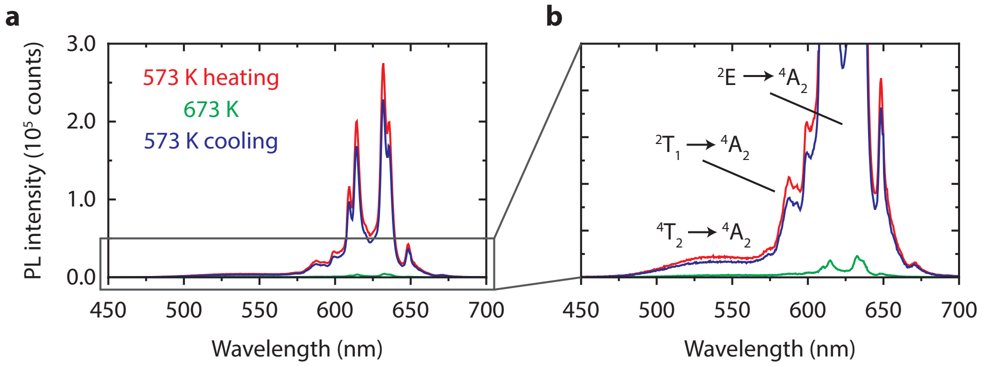

Figure S7 High temperature $^4T_2 \rightarrow {}^4A_2$ and $^2T_1 \rightarrow {}^4A_2$ emission in $K_2SiF_6:Mn^{4+}$ (**a**) PL emission spectra of $K_2SiF_6:Mn^{4+}$ at T = 573 K (red), at T = 673 K (green) and at T = 573 K upon cooling from 673 K (blue). The excitation wavelength is 360 nm. (**b**) Zoom of the gray area in (**a**). The intense emission peaks around 620 nm are assigned to the Mn^{4+} $^2E \rightarrow {}^4A_2$ transition, while the weaker emissions at wavelengths shorter than 600 nm are assigned to the Mn^{4+} $^4T_2 \rightarrow {}^4A_2$ and $^2T_1 \rightarrow {}^4A_2$ transitions.

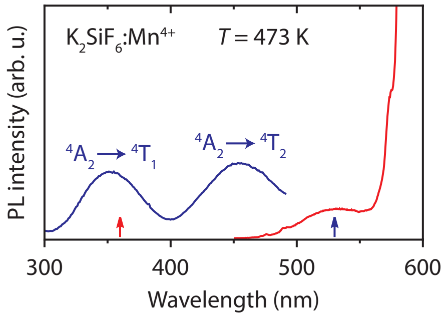

Figure S8 PL excitation (blue, λ_{em} = 530 nm) and emission (red, λ_{exc} = 360 nm) spectra of $K_2SiF_6:Mn^{4+}$ at T = 473 K. The red and blue arrows indicate the excitation and emission wavelengths used for recording the spectra, respectively. The excitation spectrum (blue) of the weak emission band centered at 530 nm consists of two excitation bands that are assigned to the $^4A_2 \rightarrow {}^4T_1$ and $^4A_2 \rightarrow {}^4T_2$ transitions of Mn^{4+}.

9. Bandwidth of $^4A_2 \rightarrow {}^4T_2$ excitation band in Mn^{4+}-doped fluorides

To investigate the variation in the offset Δ*R* for Mn^{4+}-doped fluorides, we compare the bandwidth of the $^4A_2 \rightarrow {}^4T_2$ excitation band in K_2TiF_6:Mn^{4+}, K_2SiF_6:Mn^{4+} and Cs_2HfF_6:Mn^{4+}. The width of the $^4A_2 \rightarrow {}^4T_2$ excitation band is controlled by the displacement of the $^4T_2$ state and therefore gives a good indication of Δ*R*. In Figure S9 it is observed that the full width at half maximum intensity (fwhm) of the $^4A_2 \rightarrow {}^4T_2$ band increases from 3104 cm^{-1} in K_2TiF_6:Mn^{4+} to 3251 cm^{-1} in Cs_2HfF_6:Mn^{4+}. This shows that the width of the $^4A_2 \rightarrow {}^4T_2$ excitation band, and thereby Δ*R*, varies per fluoride host lattice. The energy difference between the fwhm values is however small compared to the difference in $^4A_2 \rightarrow {}^4T_2$ energy, which indicates that the $^4T_2$ level energy is more important for the quenching temperature.

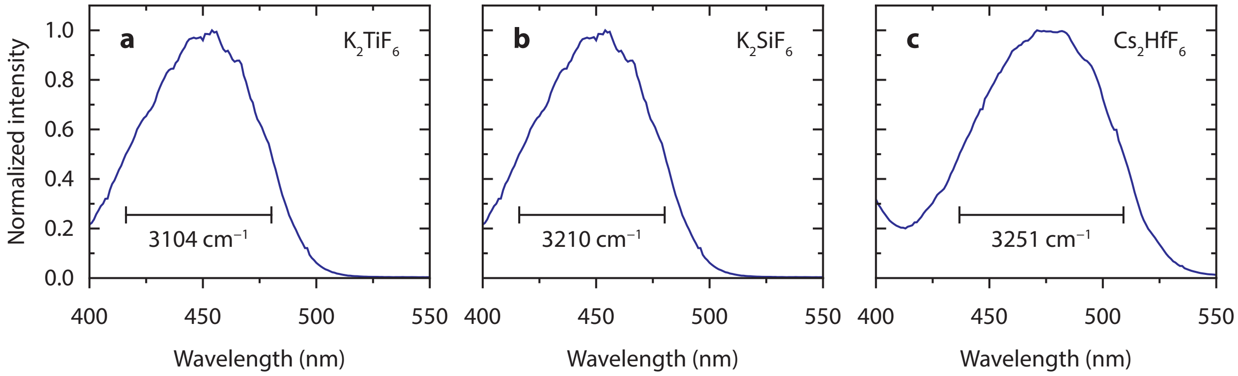

Figure S9 PL spectra of the $^4A_2 \rightarrow {}^4T_2$ excitation band in (**a**) K_2TiF_6:Mn^{4+}(0.8%), (**b**) K_2SiF_6:Mn^{4+} (BR301-C commercial phosphor from Mitsubishi Chemical, Japan) and (**c**) Cs_2HfF_6:Mn^{4+} (2.3%). The data of Cs_2HfF_6:Mn^{4+} was obtained from Ref. 8. The horizontal bars indicate the full width at half maximum intensity (fwhm), which is 3104, 3210 and 3251 cm^{-1} for K_2TiF_6:Mn^{4+}, K_2SiF_6:Mn^{4+} and Cs_2HfF_6:Mn^{4+}, respectively.

10. Influence of host lattice on Mn^{4+} luminescence quenching temperature

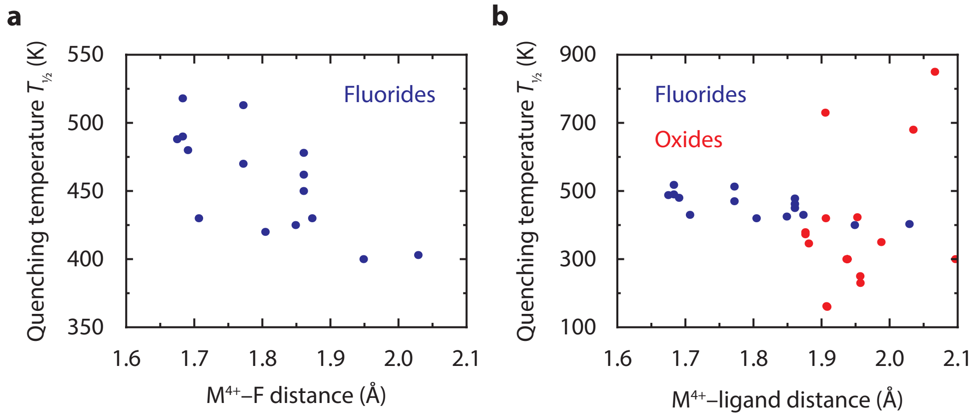

Figure S10 (**a**) Quenching temperature $T_{½}$ as a function of the average distance between the M^{4+} ion and surrounding F^- ligands for Mn^{4+}-doped fluoride phosphors. (**b**) Dependence of the quenching temperature $T_{½}$ on the average M^{4+}–ligand distance for Mn^{4+}-doped fluorides (blue dots) and Mn^{4+}-doped oxides (red dots). The data displayed in this figure is also listed in Table S2.

Table S2 Quenching temperature $T_{½}$ and M^{4+}–ligand distance of Mn^{4+}-doped phosphors. The $T_{½}$ values were obtained from the given references. The M^{4+}–ligand distances were obtained from crystal structures reported by the International Centre for Diffraction Data (ICDD).

Host lattice	M^{4+}–ligand distance (Å)	$T_{½}$ (K)	References
K_2TiF_6	1.861	462	This work
K_2SiF_6	1.683	518	This work
K_2SiF_6	1.683	490	[10]
K_2GeF_6	1.772	470	[10]
K_2TiF_6	1.861	450	[10]
K_2TiF_6	1.861	478	[11]
Na_2SiF_6	1.675	488	[30]
Rb_2SiF_6	1.690	480	[31]
Rb_2GeF_6	1.772	513	[32]
Cs_2GeF_6	1.804	420	[33]
Cs_2SiF_6	1.873	430	[33]
Cs_2HfF_6	2.029	403	[8]
$BaSiF_6$	1.706	430	[34]
$BaSnF_6$	1.949	400	[35]
$BaTiF_6$	1.849	425	[36]
Mg_4GeO_6	1.905	730	[12]
$K_2Ge_4O_9$	1.876	373	[15]
$K_2Ge_4O_9$	1.908	160	[16]
$K_2Ge_4O_9$ (site 1)	1.876	379	[16]
$K_2Ge_4O_9$ (site 2)	1.907	162	[16]
$Rb_2Ge_4O_9$ (site 1)	1.881	346	[16]
$Y_2Mg_3Ge_3O_{12}$	2.066	850	[17]
$La_3GaGe_5O_{16}$	1.906	420	[18]
La_2ZnTiO_6	1.957	230	[19]
La_2MgTiO_6	1.957	250	[19]
$CaZrO_3$	2.096	300	[14,20]
$Mg_6As_2O_{11}$	2.035	680	[21,22]
$Y_3Al_5O_{12}$	1.938	300	[23]
$Y_3Al_5O_{12}$	1.938	300	[24]
$Sr_4Al_{14}O_{25}$	1.952	423	[25]
$SrLaAlO_4$	1.936	300	[26]
$LiGa_5O_8$	1.988	350	[3]

11. Spectral overlap between Mn^{4+} emission and excitation at 4 K

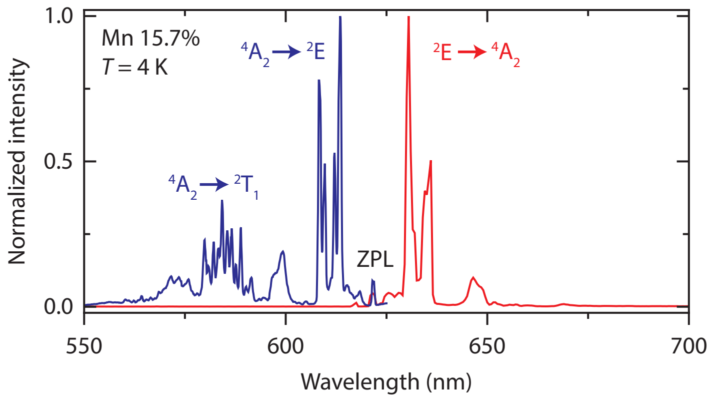

Figure S11 PL excitation (blue, λ_{em} = 636 nm) and emission (red, λ_{exc} = 450 nm) spectra of $K_2TiF_6:Mn^{4+}$ (15.7%) at T = 4 K. For the excitation spectrum only the spectral region of the $^4A_2 \rightarrow {}^2T_1$ and $^4A_2 \rightarrow {}^2E$ transitions is shown.

12. Energy transfer between Mn^{4+} ions

The results presented in Figure 5 show that concentration quenching via energy migration is weak in $K_2TiF_6:Mn^{4+}$. We attribute this to the fact that energy transfer between Mn^{4+} neighbors probably has to occur via exchange interaction[14,37]. The very small oscillator strength of the zero-phonon line prevents efficient resonant energy transfer via dipole-dipole interaction. Energy transfer via exchange interaction (wavefunction overlap) is possible but only active for very small (<5 Å) distances between the Mn^{4+} ions and is therefore limited to transfer between nearest neighbors (nearest neighbor distance is 4.7 Å in K_2TiF_6). With a Mn^{4+} doping concentration of e.g., 5%, most Mn^{4+} ions will not have Mn^{4+} neighbors within a distance of 5 Å[38]. Energy transfer between Mn^{4+} ions will therefore be very inefficient and as a consequence energy migration to quenching sites is limited in $K_2TiF_6:Mn^{4+}$. This situation will also apply to other Mn^{4+}-doped fluoride phosphors. We therefore expect concentration quenching by energy migration in general not to be an issue for the use of Mn^{4+}-doped fluorides in w-LEDs. The quenching that is observed for higher Mn^{4+} concentrations is explained by an increase in the amount of quenching centers with increasing manganese concentration.

References

1. Bachmann V, Ronda C, Meijerink A. Temperature quenching of yellow Ce^{3+} luminescence in YAG:Ce. *Chem Mater* 2009; **21**: 2077–2084.
2. Donegan JF, Glynn TJ, Imbusch GF, Remeika JP. Luminescence and fluorescence line narrowing studies of $Y_3Al_5O_{12}:Mn^{4+}$. *J Lumin* 1986; **36**: 93–100.
3. Da Fonseca RJM, Abritta T. Radiative and nonradiative processes in $LiGa_5O_8:Mn^{4+}$. *Phys B Condens Matter* 1993; **190**: 327–332.
4. Brenier A, Suchocki A, Pedrini C, Boulon G, Madej C. Spectroscopy of Mn^{4+}-doped Ca-substituted gadolinium gallium garnet. *Phys Rev B* 1992; **46**: 3219–3227.
5. Suchocki A, Allen JD, Powell RC, Loiacono GM. Spectroscopy and four-wave mixing in $Li_4Ge_5O_{12}:Mn^{4+}$ crystals. *Phys Rev B* 1987; **36**: 6729–6734.
6. de Jong M, Seijo L, Meijerink A, Rabouw FT. Resolving the ambiguity in the relation between Stokes shift and Huang–Rhys parameter. *Phys Chem Chem Phys* 2015; **17**: 16959–16969.
7. Forrest IW, Lane AP. Single-crystal polarized infrared and Raman spectra and normal-coordinate analysis of some Group 4 complex hexafluorometalates. *Inorg Chem* 1976; **15**: 265–269.
8. Senden T, van Harten EJ, Meijerink A. Synthesis and narrow red luminescence of $Cs_2HfF_6:Mn^{4+}$, a new phosphor for warm white LEDs. *J Lumin* 2018; **194**: 131–138.
9. Henderson B, Imbusch GF. *Optical Spectroscopy of Inorganic Solids*. Oxford University Press: Oxford, 1989.
10. Paulusz AG. Efficient Mn(IV) emission in fluorine coordination. *J Electrochem Soc* 1973; **120**: 942–947.
11. Zhu H, Lin CC, Luo W, Shu S, Liu Z *et al.* Highly efficient non-rare-earth red emitting phosphor for warm white light-emitting diodes. *Nat Commun* 2014; **5**: 4312.
12. Thorington L. Temperature dependence of the emission of an improved manganese-activated magnesium germanate phosphor. *J Opt Soc Am* 1950; **40**: 579–583.
13. Kemeny G, Haake CH. Activator center in magnesium fluorogermanate phosphors. *J Chem Phys* 1960; **33**: 783–789.
14. Blasse G, Grabmaier BC. *Luminescent Materials*. Springer-Verlag: Berlin, 1994.
15. Li P, Wondraczek L, Peng M, Zhang Q. Tuning Mn^{4+} red photoluminescence in $(K,Rb)_2Ge_4O_9:Mn^{4+}$ solid solutions

by partial alkali substitution. *J Am Ceram Soc* 2016; **99**: 3376–3381.
16. Baur F, Jüstel T. Dependence of the optical properties of Mn^{4+} activated $A_2Ge_4O_9$ (A = K, Rb) on temperature and chemical environment. *J Lumin* 2016; **177**: 354–360.
17. Jansen T, Gorobez J, Kirm M, Brik MG, Vielhauer S *et al.* Narrow band deep red photoluminescence of $Y_2Mg_3Ge_3O_{12}:Mn^{4+},Li^+$ inverse garnet for high power phosphor converted LEDs. *ECS J Solid State Sci Technol* 2018; **7**: R3086–R3092.
18. Zhang S, Hu Y, Duan H, Chen L, Fu Y *et al.* Novel $La_3GaGe_5O_{16}:Mn^{4+}$ based deep red phosphor: a potential color converter for warm white light. *RSC Adv* 2015; **5**: 90499–90507.
19. Takeda Y, Kato H, Kobayashi M, Kobayashi H, Kakihana M. Photoluminescence properties of Mn^{4+}-activated perovskite-type titanates, La_2MTiO_6:Mn4+ (M = Mg and Zn). *Chem Lett* 2015; **44**: 1541–1543.
20. Blasse G, de Korte PHM. The luminescence of tetravalent manganese in $CaZrO_3$:Mn. *J Inorg Nucl Chem* 1981; **43**: 1505–1506.
21. Yen WM, Shionoya S, Yamamoto H. *Phosphor handbook*. CRC Press, 2007.
22. Travniçek M, Kröger FA, Botden TPJ, Zalm P. The luminescence of basic magnesium arsenate activated by manganese. *Physica* 1952; **18**: 33–42.
23. Chen D, Zhou Y, Zhong J. A review on Mn^{4+} activators in solids for warm white light-emitting diodes. *RSC Adv* 2016; **6**: 86285–86296.
24. Riseberg LA, Weber MJ. Spectrum and anomalous temperature dependence of the $^2E \rightarrow {}^4A_2$ emission of $Y_3Al_5O_{12}:Mn^{4+}$. *Solid State Commun* 1971; **9**: 791–794.
25. Peng M, Yin X, Tanner PA, Brik MG, Li P. Site occupancy preference, enhancement mechanism, and thermal resistance of Mn^{4+} red luminescence in $Sr_4Al_{14}O_{25}:Mn^{4+}$ for warm WLEDs. *Chem Mater* 2015; **27**: 2938–2945.
26. Zhydachevskii Y, Suchocki A, Pajaczkowska A, Kłos A, Szysiak A *et al.* Spectroscopic properties of Mn^{4+} ions in $SrLaAlO_4$. *Opt Mater* 2013; **35**: 1664–1668.
27. Fonger WH, Struck CW. Temperature dependences of Cr^{3+} radiative and nonradiative transitions in ruby and emerald. *Phys Rev B* 1975; **11**: 3251–3260.
28. Malysa B, Meijerink A, Jüstel T. Temperature dependent luminescence Cr^{3+}-doped $GdAl_3(BO_3)_4$ and $YAl_3(BO_3)_4$. *J Lumin* 2016; **171**: 246–253.
29. Shen YR, Bray KL. Effect of pressure and temperature on the lifetime of Cr^{3+} in yttrium aluminum garnet. *Phys Rev B* 1997; **56**: 882–891.
30. Nguyen HD, Lin CC, Fang MH, Liu RS. Synthesis of $Na_2SiF_6:Mn^{4+}$ red phosphors for white LED applications by co-precipitation. *J Mater Chem C* 2014; **2**: 10268–10272.
31. Sakurai S, Nakamura T, Adachi S. $Rb_2SiF_6:Mn^{4+}$ and $Rb_2TiF_6:Mn^{4+}$ red-emitting phosphors. *ECS J Solid State Sci Technol* 2016; **5**: R206–R210.
32. Wu WL, Fang MH, Zhou W, Lesniewski T, Mahlik S *et al.* High color rendering index of $Rb_2GeF_6:Mn^{4+}$ for light-emitting diodes. *Chem Mater* 2017; **29**: 935–939.
33. Arai Y, Adachi S. Optical transitions and internal vibronic frequencies of MnF_6^{2-} ions in Cs_2SiF_6 and Cs_2GeF_6 red phosphors. *J Electrochem Soc* 2011; **158**: J179–J183.
34. Sekiguchi D, Nara J, Adachi S. Photoluminescence and Raman scattering spectroscopies of $BaSiF_6:Mn^{4+}$ red phosphor. *J Appl Phys* 2013; **113**: 183516.
35. Hoshino R, Nakamura T, Adachi S. Synthesis and photoluminescence properties of $BaSnF_6:Mn^{4+}$ red phosphor. *ECS J Solid State Sci Technol* 2016; **5**: R37–R43.
36. Mo G, Wang W, Wang K, Wen G, Zhu M, Wang J. Deep red $BaTiF_4:Mn^{4+}$ phosphor: synthesis, optical properties and application for warm WLED devices. *J Mater Sci Mater Electron* 2017; **28**: 8155–8159.
37. Birgeneau RJ. Mechanisms of energy transport in ruby. *J Chem Phys* 1969; **50**: 4282–4287.
38. Göbel O. Rerefinement of $K_2[TiF_6]$. *Acta Crystallogr Sect C* 2000; **56**: 521–522.
